# Supplementary material for: High-volume hybridoma sequencing on the NeuroMabSeq platform enables efficient generation of recombinant monoclonal antibodies and scFvs for neuroscience research
Source: Sci Rep. 2023 Sep 27;13:16200. doi: 10.1038/s41598-023-43233-4 (PMC10533561; doi:10.1038/s41598-023-43233-4)
Supplement: Supplementary file 1 — Supplementary Information. [file 41598_2023_43233_MOESM1_ESM.pdf]

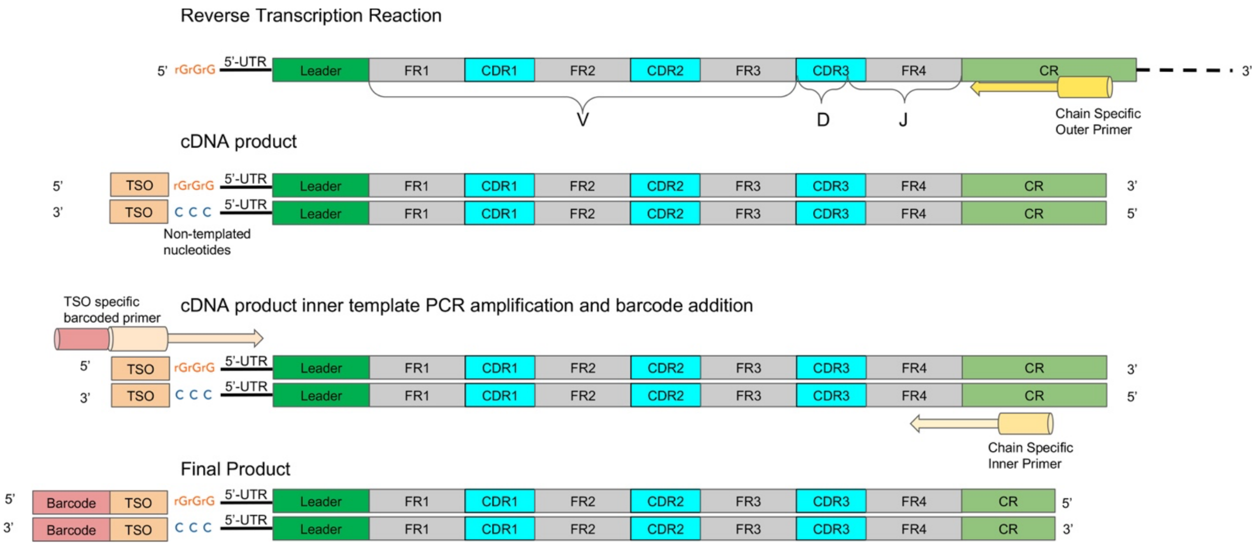


**Supplementary Figure 1**. cDNA synthesis and PCR amplification overview. Schematic shows location of nested chain-specific primers used for cDNA synthesis (outer) and PCR amplification (inner). Also shown is the location of the template switching oligonucleotide. Schematic depicts the synthesis and amplification of VH domain cDNA, analogous steps are used for VL domains. FR: framework regions; CDR: complementarity determining regions; CR: constant region.

**Supplementary Table 1. List of primers and barcodes.**

| Oligonucleotide | Vendor | Cat # | Sequence |
|---|---|---|---|
| RT Chain Specific Outer Primer mix | | | |
| RT-mIGK | IDT | 271038654 | TTGTCGTTCACTGCCATCAAT*C |
| RT-mIGHG | IDT | 271038655 | AGCTGGGAAGGTGTGCACA*C |
| RT-mIGL | IDT | 271038656 | GGGGTACCATCTACCTTCCA*G |
| RT-mIGHG3-NEW | IDT | 271038657 | GTACAGATGAGACTGTGCGCACA*C |
| | | | |
| TSO-short | IDT | 408473058 | /5Biosg/AAGCAGTGGTATCAACGCAGAGTACA TrGrGrG |
| | | | |
| PCR Chain Specific Reverse Inner Primer Mix | | | |
| 10for-mIgh3 | IDT | 270929982 | GGATAGACAGATGGGGCTGTTGTTGTAG*C |
| PCR-R-mIGL | IDT | 270929983 | ATCGTACACACCAGTGTGG*C |
| 6-REV-LC | IDT | 270929984 | GGATACAGTTGGTGCAGCAT*C |
| 10-REV-HC1 | IDT | 270929985 | ATAGACAGATGGGGGTGTCGTTTTGG*C |

| F Primer, ISPCR (Barcoded) PCR-SMART indices | Sequence |
|---|---|
| 01-SMARTindex | CAGCGTCAGTGGTATCAACGCAGAGTACA |
| 02-SMARTindex | GATCACCAGTGGTATCAACGCAGAGTACA |
| 03-SMARTindex | ACCAGTCAGTGGTATCAACGCAGAGTACA |
| 04-SMARTindex | TGCACGCAGTGGTATCAACGCAGAGTACA |
| 05-SMARTindex | ACATTACAGTGGTATCAACGCAGAGTACA |
| 06-SMARTindex | GTGTAGCAGTGGTATCAACGCAGAGTACA |
| 07-SMARTindex | CTAGTCCAGTGGTATCAACGCAGAGTACA |
| 08-SMARTindex | TGTGCACAGTGGTATCAACGCAGAGTACA |
| 09-SMARTindex | TCAGGAACAGTGGTATCAACGCAGAGTACA |
| 10-SMARTindex | CGGTTAACAGTGGTATCAACGCAGAGTACA |
| 11-SMARTindex | TTAACTACAGTGGTATCAACGCAGAGTACA |
| 12-SMARTindex | ATGAACACAGTGGTATCAACGCAGAGTACA |
| 13-SMARTindex | CCTAAGACAGTGGTATCAACGCAGAGTACA |
| 14-SMARTindex | AATCCGACAGTGGTATCAACGCAGAGTACA |
| 15-SMARTindex | GGCTGCACAGTGGTATCAACGCAGAGTACA |
| 16-SMARTindex | TACCTTACAGTGGTATCAACGCAGAGTACA |
| 17-SMARTindex | TCTTAATCAGTGGTATCAACGCAGAGTACA |
| 18-SMARTindex | GTCAGGTCAGTGGTATCAACGCAGAGTACA |
| 19-SMARTindex | ATACTGTCAGTGGTATCAACGCAGAGTACA |
| 20-SMARTindex | TATGTCTCAGTGGTATCAACGCAGAGTACA |
| 21-SMARTindex | GAGTCCTCAGTGGTATCAACGCAGAGTACA |
| 22-SMARTindex | GGAGGTTCAGTGGTATCAACGCAGAGTACA |
| 23-SMARTindex | CACACTTCAGTGGTATCAACGCAGAGTACA |
| 24-SMARTindex | CCGCAATCAGTGGTATCAACGCAGAGTACA |
| 25-SMARTindex | TTTATGCAGTGGTATCAACGCAGAGTACA |
| 26-SMARTindex | AACGCCCAGTGGTATCAACGCAGAGTACA |
| 27-SMARTindex | CAAGCACAGTGGTATCAACGCAGAGTACA |
| 28-SMARTindex | GCTCGACAGTGGTATCAACGCAGAGTACA |
| 29-SMARTindex | GCGAATCAGTGGTATCAACGCAGAGTACA |
| 30-SMARTindex | TGGATTCAGTGGTATCAACGCAGAGTACA |
| 31-SMARTindex | ACCTACCAGTGGTATCAACGCAGAGTACA |
| 32-SMARTindex | CGAAGGCAGTGGTATCAACGCAGAGTACA |
| 33-SMARTindex | AGATAGAACAGTGGTATCAACGCAGAGTACA |
| 34-SMARTindex | TTGGTAAACAGTGGTATCAACGCAGAGTACA |

| | |
|---|---|
| 35-SMARTindex | GTTACCAACAGTGGTATCAACGCAGAGTACA |
| 36-SMARTindex | CGCAACAACAGTGGTATCAACGCAGAGTACA |
| 37-SMARTindex | TGGCGAAACAGTGGTATCAACGCAGAGTACA |
| 38-SMARTindex | ACCGTGAACAGTGGTATCAACGCAGAGTACA |
| 39-SMARTindex | CAACAGAACAGTGGTATCAACGCAGAGTACA |
| 40-SMARTindex | GATTGTAACAGTGGTATCAACGCAGAGTACA |
| 41-SMARTindex | CTCTCGATCAGTGGTATCAACGCAGAGTACA |
| 42-SMARTindex | TGACACATCAGTGGTATCAACGCAGAGTACA |
| 43-SMARTindex | AAGACAATCAGTGGTATCAACGCAGAGTACA |
| 44-SMARTindex | ACAGATATCAGTGGTATCAACGCAGAGTACA |
| 45-SMARTindex | TAGGCTATCAGTGGTATCAACGCAGAGTACA |
| 46-SMARTindex | CTCCATATCAGTGGTATCAACGCAGAGTACA |
| 47-SMARTindex | GCATGGATCAGTGGTATCAACGCAGAGTACA |
| 48-SMARTindex | AATAGCATCAGTGGTATCAACGCAGAGTACA |
| 49-SMARTindex | GTGCCATACAGTGGTATCAACGCAGAGTACA |
| 50-SMARTindex | TCGAGGTACAGTGGTATCAACGCAGAGTACA |
| 51-SMARTindex | CACTAATACAGTGGTATCAACGCAGAGTACA |
| 52-SMARTindex | GGTATATACAGTGGTATCAACGCAGAGTACA |
| 53-SMARTindex | CGCCTGTACAGTGGTATCAACGCAGAGTACA |
| 54-SMARTindex | AATGAATACAGTGGTATCAACGCAGAGTACA |
| 55-SMARTindex | ACAACGTACAGTGGTATCAACGCAGAGTACA |
| 56-SMARTindex | ATATCCTACAGTGGTATCAACGCAGAGTACA |
| 57-SMARTindex | AGTACTCAGTGGTATCAACGCAGAGTACA |
| 58-SMARTindex | ATAAGACAGTGGTATCAACGCAGAGTACA |
| 59-SMARTindex | GGTGAGCAGTGGTATCAACGCAGAGTACA |
| 60-SMARTindex | TTCCGCCAGTGGTATCAACGCAGAGTACA |
| 61-SMARTindex | GAAGTGCAGTGGTATCAACGCAGAGTACA |
| 62-SMARTindex | CAATGCCAGTGGTATCAACGCAGAGTACA |
| 63-SMARTindex | ACGTCTCAGTGGTATCAACGCAGAGTACA |
| 64-SMARTindex | CAGGACCAGTGGTATCAACGCAGAGTACA |
| 65-SMARTindex | AAGCTCCAGTGGTATCAACGCAGAGTACA |
| 66-SMARTindex | GACGATCAGTGGTATCAACGCAGAGTACA |
| 67-SMARTindex | TCGTTCCAGTGGTATCAACGCAGAGTACA |
| 68-SMARTindex | CCAATTCAGTGGTATCAACGCAGAGTACA |
| 69-SMARTindex | AGTTGACAGTGGTATCAACGCAGAGTACA |
| 70-SMARTindex | AACCGACAGTGGTATCAACGCAGAGTACA |
| 71-SMARTindex | CAGATGCAGTGGTATCAACGCAGAGTACA |
| 72-SMARTindex | GTAGAACAGTGGTATCAACGCAGAGTACA |
| 73-SMARTindex | GACATCACAGTGGTATCAACGCAGAGTACA |
| 74-SMARTindex | CGATCTACAGTGGTATCAACGCAGAGTACA |
| 75-SMARTindex | CGTCGCACAGTGGTATCAACGCAGAGTACA |
| 76-SMARTindex | ATGGCGACAGTGGTATCAACGCAGAGTACA |
| 77-SMARTindex | ATTGGTACAGTGGTATCAACGCAGAGTACA |
| 78-SMARTindex | GCCACAACAGTGGTATCAACGCAGAGTACA |
| 79-SMARTindex | CATCTAACAGTGGTATCAACGCAGAGTACA |
| 80-SMARTindex | AACAAGACAGTGGTATCAACGCAGAGTACA |
| 81-SMARTindex | GCAGCCTCAGTGGTATCAACGCAGAGTACA |
| 82-SMARTindex | ACTCTTTCAGTGGTATCAACGCAGAGTACA |
| 83-SMARTindex | TGCTATTCAGTGGTATCAACGCAGAGTACA |
| 84-SMARTindex | AAGTGGTCAGTGGTATCAACGCAGAGTACA |
| 85-SMARTindex | CTCATATCAGTGGTATCAACGCAGAGTACA |
| 86-SMARTindex | CCGACCTCAGTGGTATCAACGCAGAGTACA |
| 87-SMARTindex | GGCCAATCAGTGGTATCAACGCAGAGTACA |

| | |
|---|---|
| 88-SMARTindex | AGACCATCAGTGGTATCAACGCAGAGTACA |
| 89-SMARTindex | CGCGGACAGTGGTATCAACGCAGAGTACA |
| 90-SMARTindex | CCTGCTCAGTGGTATCAACGCAGAGTACA |
| 91-SMARTindex | GCGCTGCAGTGGTATCAACGCAGAGTACA |
| 92-SMARTindex | GAACCTCAGTGGTATCAACGCAGAGTACA |
| 93-SMARTindex | TTCGAGCAGTGGTATCAACGCAGAGTACA |
| 94-SMARTindex | AGAATCCAGTGGTATCAACGCAGAGTACA |
| 95-SMARTindex | AGGCATCAGTGGTATCAACGCAGAGTACA |
| 96-SMARTindex | ACACGCCAGTGGTATCAACGCAGAGTACA |

**Supplementary Table 1.** Top table. Sequences of oligonucleotides used for priming cDNA synthesis and for amplification of $V_L$ and $V_H$ domains. Bottom table. Sequences of oligonucleotides used to barcode samples.

## Supplementary Table 2. List of R-mAbs successfully cloned via sequence-based cloning.

| R-mAb | Target | R-mAb | Target | R-mAb | Target | R-mAb | Target |
|---|---|---|---|---|---|---|---|
| 1D8 | LRRK2/Dardarin-pSer910 | L114/3 | Parvalbumin | N133/35 | RGS14 | N358/68 | 5-formylcytidine |
| 1F1 | TrpC1 | L114/38 | Parvalbumin | N134/12 | Nav1.8 Na+ channel | N360A/24 | ATF4 |
| 62-3G1 | GABA(A)R, Beta2/3 | L114/81 | Parvalbumin | N138/6 | LRRK2/Dardarin, N-terminus | N363/71 | Kir6.2 K+ channel |
| 8G10 | LRRK2/Dardarin, N-terminus | L115/13 | NPY Neuropeptide Y | N140A/12 | SALM5/LRFN5 | N364/10 | Bral1 |
| A12/18 | Pan-Neurofascin (extracellular) | L117/1 | IRSp53/BAIAP2 | N141/21 | HCN3 | N364/42 | Bral1 |
| D4/11 | Kv2.1 K+ channel | L117/52 | IRSp53/BAIAP2 | N141/28 | HCN3 | N366/60 | Kir6.1 K+ channel |
| D4/154 | Kv2.1 K+ channel | L118/14 | VGAT | N142/28 | GRK6A/B | N367/51 | Shank1 and Shank3 |
| D4/40 | Kv2.1 K+ channel | L118/80 | VGAT | N143/36 | CDY1/2/L1/L2 | N367/62 | Shank3 |
| K14/39 | Kv1.2 K+ channel | L118/135 | VGAT | N143/38 | CDY1/2 | N372A/1 | Kv2.2 K+ channel |
| K19/46 | Kv1.6 K+ channel | L119/106 | NPY2R Neuropeptide Y receptor type 2 | N144/14 | 6xHis | N372B/1 | Kv2.2 K+ channel |
| K28/25 | PSD-95 + PSD-93 | L119/134* | NPY2R Neuropeptide Y receptor type 2 | N144/17 | Gamma-protocadherin-A3 | N372B/60 | Kv2.2 K+ channel |
| K28/27 | PSD-95 | L120/12 | Collybistin | N144/32 | Pan-Gamma-protocadherin-A | N372C/51 | Kv2.2 K+ channel |
| K28/38 | PSD-95 | L120/30 | Collybistin | N145/20 | Pan-GRK | N374/48 | TASK1 K+ channel |
| K28/42 | PSD-95 + PSD-93 | L121/25 | nNOS/NOS1 | N148/30 | Gamma-protocadherin-B2 | N375/67 | Kv3.3 K+ channel |
| K28/58 | PSD-95 | L121/42 | nNOS/NOS1 | N152B/23 | VDAC1 | N377/20 | MAP3K12 |
| K28/74 | PSD-95 | L121/133 | nNOS/NOS1 | N154/32 | JMJD2A | N377/83 | MAP3K12 |
| K28/86 | Pan-MAGUK | L121/136 | nNOS/NOS1 | N155/9 | Uncx | N37A/10 | Kv7.1/KCNQ1 K+ channel |
| K28/94 | PSD-95 | L122/6 | Calretinin | N158/28 | Histone H3.3 | N380/87 | CLN3 |
| K40/17 | Pan-Kvbeta K+ channel | L122/68 | Calretinin | N159/5 | Pan-Gamma-protocadherin-Constant | N382/14 | MFF |
| K47/9 | Kvbeta1.2/KCNAB1 K+ channel | L122/144 | Calretinin | N160/21 | SynDIG3/Tmem91 | N382/69 | MFF (exon 1) |
| K47/42 | Kvbeta1.2/KCNAB1 K+ channel | L123/46 | GABA(A)R, Theta | N161/20 | NKCC1 | N384/63 | Miro2 |
| K55/7* | KChIP1 K+ channel | L124/59 | Bassoon | N165/38 | LAR/PTPRF | N385/21 | Beta1-spectrin |
| K55/29 | KChIP1 K+ channel | L125/121 | Synapsin-3 | N165/43 | Pan-PTPR | N388A/27 | Ankyrin-R/G |
| K55/82 | Pan-KChIP K+ channel | L125/129 | Pan-Synapsin | N166A/26 | OCRL/INPP5b | N389/9 | TRAK1 |
| K56A/50 | CASK | L125/76 | SAPAP3 | N170A/26 | Neurexin-1-Beta | N390/43 | TRAK2 |
| K56A/57 | CASK | L126/93 | SAPAP3 | N173B/13 | Tafazzin | N391/68 | WNK1 |
| K57/27 | Kv4.2 K+ channel (external) | L127/8 | GAD67 | N180/41 | EAAC1 | N393/2 | Beta4-spectrin |
| K57/41 | Kv4.2 K+ channel (external) | L127/12 | GAD65/67 | N183/15 | QKI-7 | N393/76 | Beta4-spectrin |
| K60/73 | KChIP2b K+ channel | L130/1 | Tiam1 | N185/7 | NALCN | N395/68 | Navbeta2 Na+ channel |
| K60/87** | KChIP2b K+ channel | L131/17 | SPHKAP | N186/29 | Dopamine D2 receptor | N396/29 | Navbeta3 Na+ channel |
| K65/35 | CASPR/Neurexin IV | L131/20 | SPHKAP | N191/7 | Frataxin | N397/19 | Nav1.5 Na+ channel |
| K65A/2 | CASPR/Neurexin IV | L131/27 | SPHKAP | N192/12 | Gs protein, alpha subunit | N398A/34 | GABA(A)R, Alpha4 |
| K66/27 | KChIP3 K+ channel | L132/18 | Cav1.2 Ca2+ channel pS1928 | N194/11 | QKI-7b | N399/19 | GABA(A)R, Alpha2 |
| K66/59 | KChIP3 K+ channel | L132/37 | Cav1.2 Ca2+ channel pS1928 | N202/7 | Fig4/Sac3 | N400/24 | CLN6 |
| K67/11 | CASPR2 | N1/12 | KCC2 | N206A/8 | GFAP | N402/13 | Thyroid hormone receptor beta1 |

| | | | | | | | |
|---|---|---|---|---|---|---|---|
| K67/25 | CASPR2 | N4/15 | PINK1 | N207/27 | LRP4 (extracellular) | N402/46 | Thyroid hormone receptor beta1 |
| K69/3* | Nav1.2 Na+ channel | N4/49 | PINK1 | N209C/35 | LRRTM2 | N403/63 | Pan-Thyroid hormone receptor |
| K69/33 | Nav1.2 Na+ channel | N6/38 | VAChT | N210/5 | PhyH/PAHX | N405/74 | Navbeta1 Na+ channel |
| K73/20 | Contactin/F3 | N10/7 | Cavbeta4 Ca2+ channel | N212/7 | TRIP8b (constant) | N406/47 | Clcn5 |
| K74/53 | Nav1.1 Na+ channel | N15/4 | TrpV3 | N212A/34 | TRIP8b (exon 1b) | N408/79 | Npas4 |
| K75/18 | Kv4.3 K+ channel | N15/39 | TrpV3 | N219/5 | RBM17/SPF45 | N410/17 | Kv3.2 K+ channel |
| K75/30 | Kv4.3 K+ channel | N18/28 | PSD-93/Chapsyn-110 | N221/12 | TrpV1 | N411/51 | Arx |
| K78/29 | SK2 K+ channel | N18/30 | PSD-93/Chapsyn-110 | N221/17 | TrpV1 | N414/25 | NCKX4 |
| K87A/10 | Nav1.6 Na+ channel | N19/2 | SAP102 | N225A/10 | FGF13/FHF2, B isoform | N416/57 | GluN3A/NR3A glutamate receptor |
| K89/41 | Kv2.1 K+ channel | N22/21 | Shank1 | N227/21 | MECP2 | N420/24 | EAAC1 |
| K96/7 | Pancortin | N23B/6 | Shank2 | N228A/16 | Lhx6.1 | N421A/85 | ANO5/TMEM16E |
| L6/48 | Slo1/BKAlpha maxi-K+ channel | N25/35 | Kir2.3 K+ channel | N229A/32 | GABA(A)R, Alpha6 | N422/18 | GluN2C/NR2C glutamate receptor |
| L6/60 | Slo1/BKAlpha maxi-K+ channel | N26A/23 | Kv7.2/KCNQ2 K+ channel | N231B/34 | LRRK2/Dardarin, N3 (non-mouse-reactive) | N423/75 | KChIP4 K+ channel |
| L21/32 | GluA2/GluR2 glutamate receptor | N38/8 | Cav1.3 Ca2+ channel | N232/9 | PEX7 | N424/45 | Glycine receptor Alpha3 |
| L23/27 | Kv1.3 K+ channel | N39B/8 | GIT1 | N233/8 | PEX6 | N424/48* | Glycine receptor Alpha3L |
| L24/1 | IP3 receptor, type 1 | N43/6 | Kv7.4/KCNQ4 K+ channel | N238/29 | SAPAP1 | N425/45 | VAChT |
| L24/11 | IP3 receptor, type 1 | N46/30 | ADAM22 (cytoplasmic) | N238/30 | SAPAP1 | N428/12 | WRN |
| L24/18 | IP3 receptor, type 1 | N49A/21 | NGL-1/LRRC4C | N238/31 | SAPAP1/2 | N429/19 | ANO6/TMEM16F |
| L24/19 | IP3 receptor, type 1 | N50/36 | NGL-2/LRRC4 | N241A/34 | LRRK2/Dardarin, C-terminus | N431/64 | GABA(A)R, Pi |
| L24/21 | IP3 receptor, type 1 | N52B/27 | SALM2/LRFN1 | N241A/72 | LRRK2/Dardarin, C-terminus | N432/21 | VSP |
| L28/36 | Kv4.2 K+ channel | N53/32 | BKbeta2 K+ channel | N245/1 | TARPGamma2/Stargazin | N432/63 | VSP |
| L45/30 | SynCAM1 | N55/10 | Cav3.2 Ca2+ channel | N245/36 | TARPGamma2/4/8 | N440/21 | VMAT1 |
| L48A/9 | Cav1.3 Ca2+ channel | N56/9 | S-tag | N245/44 | Thioredoxin | N440/61 | VMAT1 |
| L48A/29 | Cav1.3 Ca2+ channel | N56/21 | FGF14/FHF4 | N250/21 | DNAH7 | N441/35 | ADAM11 |
| L48A/31 | Cav1.3 Ca2+ channel | N57/2 | ADAM22 (extracellular) | N251/14 | DNAH1 | N442/28 | Alg13 |
| L57/23 | Cav1.2/1.3 Ca2+ channel | N59/20 | GluN2B/NR2B glutamate receptor | N253/32 | Notch1 | N444/63 | AGPS neo-epitope |
| L57/46 | Cav1.2/1.3 Ca2+ channel | N64A/36 | TrpC7 | N255/38 | Nav1.4 Na+ channel | N445/27 | TRAAK K+ channel |
| L57/47 | Cav1.2/1.3 Ca2+ channel | N67/15 | TrpC5 | N263/31 | Cav1.2 Ca2+ channel | N446/80 | CELF4/BRUNOL4 |
| L58A/6 | Kv2.1 K+ channel | N68/6 | Nav1.7 Na+ channel | N270/47 | Synaptotagmin-6 | N447/24 | Kv11.3 K+ channel |
| L61C/30 | Kv2.1 K+ channel | N71/37 | HCN2 | N271/44 | ASIC1 | N448/88 | Kv8.2 K+ channel |
| L62/29 | Copper ATPase 2 (Wilson's disease protein) | N72/16 | Kv3.4 K+ channel | N274/8 | Synaptotagmin-5 | N449/73 | VMAT2 |
| L64/32 | Kv1.2 K+ channel | N74/25 | TrpM7 | N275/14 | Synaptotagmin-7 | N450/53* | BDNF |
| L71/5 | Kv1.4 K+ channel (extracellular) | N75/3 | mGluR1/5 (Group I) glutamate receptor | N276A/15 | Synaptotagmin-9 | N451/73 | ICK |
| L71/22 | Kv1.4 K+ channel (extracellular) | N75/33 | mGluR1/5 (Group I) glutamate receptor | N283/7 | Lgi1 | N452/30 | GABA(A)R, Gamma2L/S |
| L76/36 | Kv1.2 K+ channel | N77/15 | TrpC4 | N286/74 | Zebrafish PSD Marker | N452/69 | GABA(A)R, Gamma2L |

| | | | | | | | |
|---|---|---|---|---|---|---|---|
| L80/21 | Kv2.1 K+ channel | N81/2 | GABA(B)R2 | N290B/25 | ARHGAP4 | N452/73 | GABA(A)R, Gamma2L/S |
| L83/11 | Kv2.1 K+ channel | N86/20 | GFP | N294A/10 | Brevican | N452/81 | GABA(A)R, Gamma2L/S |
| L83/81 | Kv2.1 K+ channel | N86/44 | GFP | N294A/6 | Brevican | N454/91 | ANO3/TMEM16C |
| L86/2 | AMIGO-1 | N86/8 | GFP | N295B/54 | Arl13b | N455/15 | Kir3.3 K+ channel |
| L86/14 | AMIGO-1 | N87/25 | GABA(A)R, Beta3 | N302/10 | BBS3/Arl6 | N456/39 | Proser1 |
| L86/33* | AMIGO-1 | N92/14 | FGF11/FHF3 | N304B/115 | BBS5 | N458/10 | Kv6.4 K+ channel |
| L86/36 | AMIGO-1 | N95/35 | GABA(A)R, Alpha1 | N307/12 | Histone H3-acetyl-Lys56 | N459/84 | SAPAP2 |
| L100/1 | Kv2.1 K+ channel pS586 | N98/47 | Neuroligin-4 | N308/48 | GluN1/NR1 glutamate receptor | N460/19 | Kv9.2 K+ channel |
| L102/45 | SynDIG4/Prrt1 | N100/13 | GST | N309A/21 | Histone H4-dimethyl-Arg3 | N461/19 | Kv9.1 K+ channel |
| L106/4 | Gephyrin | N104/37 | SNAT1 | N312/10 | NSD1 | N463/52* | DEPDC5 |
| L106/22 | Gephyrin | N105/13 | Ankyrin-B | N319A/14 | SUR2A | N465/11 | Kir2.4 K+ channel |
| L106/23 | Gephyrin | N105/17 | Ankyrin-B | N323A/31 | SUR1 and SUR2B | N467/1 | TRESK potassium channel |
| L106/83 | Gephyrin | N106/20 | Ankyrin-G | N324/2 | Rem2 | N468/37 | THIK-2 potassium channel |
| L107/39 | Neuroligin-2 | N106/36* | Ankyrin-G | N325B/65 | THAP1 | N470/22 | TRPM6 |
| L107/90* | Neuroligin-2 | N106/65 | Ankyrin-G (staining) | N326D/2 | REEP2 | N471/27 | Kv1.7 K+ channel |
| L107/93 | Neuroligin-2 | N112/16 | Kir2.1 K+ channel | N326D/13 | REEP | N472/88 | MiRP4 K+ channel |
| L107/95 | Neuroligin-2 | N112B/14 | Kir2.1 K+ channel | N327/95 | GluN2A/NR2A glutamate receptor | N473/36 | Cavbeta3 Ca2+ channel |
| L109/39 | Calbindin | N112B/29 | Kir2.1 K+ channel | N327A/38 | GluN2A/NR2A glutamate receptor | N476/9 | ZIP3 |
| L109/57 | Calbindin | N119/44 | MPP8 | N330A/80 | Alpha-2C adrenergic receptor | N479/107 | VAPA/B |
| L109/62 | Calbindin | N121A/1 | Pannexin-2 | N332B/15 | BAF53b | N479/12 | VAPA |
| L113/13 | Homer1L | N123/19 | Histone H3-pThr11 | N336B/83 | BAF53a | N479/22 | VAPA |
| L113/26 | Homer1L | N123/48 | Histone H3-pThr11 | N341/12 | LRRK1 | N483/26 | Rufy3 |
| L113/27 | Homer1L | N125/10 | Thorase/Atad1 | N343/26 | NrCAM | N483/84 | Rufy3 |
| L113/28 | Homer1L | N126B/31 | Neuregulin-CRD (Cysteine-rich domain, Type III) | N345/51 | REEP1 | N483/126 | Rufy3 |
| L113/29 | Homer1L | N128A/2 | Haspin/GSG2 | N347/42 | ATAT1 | N485/22 | Prrt2 |
| L113/44 | Homer1L | N128A/4 | Maltose binding protein (MBP) | N349/96 | Foxi2 | N486/25 | c-Fos |
| L113/62 | Homer1L/S | N129/15 | Mad3 (human) | N356/9 | SVOP | N486/32 | c-Fos |
| L113/130 | Homer1L/S | N132A/12 | KLH | N357/6* | PINK1 | N486/76 | c-Fos |

**Supplementary Table 2.** List of R-mAbs and their targets that were successfully cloned into the IgG2a mammalian expression plasmid using the sequence-based Gibson Assembly cloning method. * cloned from hybridomas with more than two $V_L$ sequences. ** cloned from a hybridoma with two $V_L$ and two $V_H$ sequences.

## Supplementary Table 3. List of R-mAbs successfully transferred to IgG subclass-switched expression plasmids.

| Transferred to IgG2b Expression Plasmid | | | | Transferred to IgG1 Expression Plasmid | |
|---|---|---|---|---|---|
| R-mAb | Target | R-mAb | Target | R-mAb | Target |
| A12/18 | Pan-Neurofascin (extracellular) | N59/20 | GluN2B/NR2B glutamate receptor | A12/18 | Pan-Neurofascin (extracellular) |
| K7/45 | Kv1.5 K+ channel | N59/36 | GluN2B/NR2B glutamate receptor | K20/78 | Kv1.1 K+ channel |
| K13/31 | Kv1.4 K+ channel | N68/6 | Nav1.7 Na+ channel | K28/43 | PSD-95 |
| K14/16 | Kv1.2 K+ channel | N69/46 | Shank3 | K57/1 | Kv4.2 K+ channel (external) |
| K17/70 | Kvbeta2 K+ channel | N70/28 | HCN1 | K65/35 | CASPR/Neurexin IV |
| K20/78 | Kv1.1 K+ channel | N76/8 | Ataxin-1, 11NQ | K75/41 | Kv4.3 K+ channel |
| K28/43 | PSD-95 | N86/38 | GFP | L108/92 | VIP |
| K39/25 | Kv2.1 K+ channel (external) | N86/8 | GFP | L109/39 | Calbindin |
| K57/1 | Kv4.2 K+ channel (external) | N97A/31 | Neuroligin-1 | L113/130 | Homer1L/S |
| K64/15 | SAP97 | N106/36 | Ankyrin-G | L113/27 | Homer1L |
| K65/35 | CASPR/Neurexin IV | N110/29 | Neuroligin-3 | L118/80 | VGAT |
| K66/38 | KChIP3 K+ channel | N112B/14 | Kir2.1 K+ channel | L122/6 | Calretinin |
| K74/71 | Nav1.1 Na+ channel | N114/10 | HCN4 | L122/68 | Calretinin |
| K75/41 | Kv4.3 K+ channel | N133/21 | RGS14 | L127/12 | GAD65 + GAD67 |
| K89/34 | Kv2.1 K+ channel | N133/35 | RGS14 | L21/32 | GluA2/GluR2 glutamate receptor |
| L6/60 | Slo1/BKAlpha maxi-K+ channel | N134/12 | Nav1.8 Na+ channel | L6/60 | Slo1/BKAlpha maxi-K+ channel |
| L21/32 | GluA2/GluR2 glutamate receptor | N149/25 | Olig1 | L86/33 | AMIGO-1 |
| L28/4 | Kv4.2 K+ channel | N151/3 | GABA(A)R, Delta | N1/12 | KCC2 |
| L57/46 | Cav1.2/1.3 Ca2+ channel | N168/6 | Navbeta4 Na+ channel | N86/8 | Nav1.7 Na+ channel |
| L60/4 | Copper ATPase 1 (Menke's disease protein) | N170A/1 | Neurexin-1-Beta | N106/36 | Ankyrin-G |
| L86/33 | AMIGO-1 | N178A/9 | Cav3.1 Ca2+ channel | N112B/14 | Kir2.1 K+ channel |
| L86/36 | AMIGO-1 | N182/17 | QKI-6 | N133/21 | RGS14 |
| L106/83 | Gephyrin | N196/16 | PARIS/ZNF746 | N134/12 | Nav1.8 Na+ channel |
| L107/39 | Neuroligin-2 | N201/35 | Iduna/RNF146 | N149/25 | Olig1 |
| L108/92 | VIP | N206A/8 | GFAP | N170A/1 | Neurexin-1-Beta |
| L109/39 | Calbindin | N212/17 | TRIP8b (constant) | N206A/8 | GFAP |
| L113/27 | Homer1L | N241A/34 | LRRK2/Dardarin, C-terminus | N241A/34 | LRRK2/Dardarin, C-terminus |
| L113/130 | Homer1L/S | N244/5 | SynCAM4 | N263/31 | Cav1.2 Ca2+ channel |
| L114/3 | Parvalbumin | N263/31 | Cav1.2 Ca2+ channel | N28/9 | VGluT1 |
| L115/13 | NPY/Neuropeptide Y | N289/16 | SUR1 | N29/29 | VGluT2 |
| L118/80 | VGAT | N295B/66 | Arl13b | N295B/66 | Arl13b |
| L122/6 | Calretinin | N308/48 | GluN1/NR1 glutamate receptor | N308/48 | GluN1/NR1 glutamate receptor |
| L122/68 | Calretinin | N327/95 | GluN2A/NR2A glutamate receptor | N327/95 | GluN2A/NR2A glutamate receptor |
| L127/8 | GAD67 | N355/1 | GluA1/GluR1 glutamate receptor | N355/1 | GluA1/GluR1 glutamate receptor |
| L127/12 | GAD65/67 | N410/17 | Kv3.2 K+ channel | N410/17 | Kv3.2 K+ channel |
| N1/12 | KCC2 | N425/45 | VAChT | N425/45 | VAChT |
| N3/26 | KCNT1/Slo2.2/Slack K+ channel | | | N52A/42 | Mortalin/GRP75 |
| N6/38 | VAChT | | | N59/20 | GluN2B/NR2B glutamate receptor |
| N7/18 | Cavbeta1 Ca2+ channel | | | N59/36 | GluN2B/NR2B glutamate receptor |
| N11/33 | KCNT2/Slo2.1/Slick K+ channel | | | N6/38 | VAChT |
| N28/9 | VGlut1 | | | N68/6 | Nav1.7 Na+ channel |
| N29/29 | VGluT2 | | | N70/28 | HCN1 |
| N52A/42 | Mortalin/GRP75 | | | N86/38 | GFP |

**Supplementary Table 3.** List of R-mAbs and their targets that were successfully transferred from the IgG2a mammalian expression plasmid into either an IgG2b and/or IgG1 mammalian expression plasmid using restriction digest/ligation-based cloning.

**Supplementary Table 4. List of scFvs successfully cloned via sequence-based cloning.**

| scFv | Target | Plasmid | Tags | scFv | Target | Plasmid | Tags |
|---|---|---|---|---|---|---|---|
| 1D8 | LRRK2/Dardarin-pSer910 | pcDNA3.4 | HA sortase His | N67/15 | TrpC5 | pcDNA3.4 | HA sortase His |
| A12/18 | Pan-Neurofascin (extracellular) | pcDNA3.1 | sortase His | N68/6 | Nav1.7 Na channel | pcDNA3.1 | sortase His |
| A12/18 | Pan-Neurofascin (extracellular) | pcDNA3.1 | HA sortase His | N69/46 | Shank3 | pcDNA3.4 | HA sortase His |
| K7/45 | Kv1.5 K channel | pcDNA3.4 | HA sortase His | N86/20 | GFP | pcDNA3.4 | HA sortase His |
| K14/16 | Kv1.2 K channel | pcDNA3.1 | HA His | N86/38 | GFP | pcDNA3.1 | HA His |
| K17/70 | Kvbeta2/KCNAB2 K channel | pcDNA3.4 | HA sortase His | N86/44 | GFP | pcDNA3.4 | HA sortase His |
| K20/78 | Kv1.1 K channel | pcDNA3.1 | sortase His | N93A/49 | GABA(B)R1 | pcDNA3.4 | HA sortase His |
| K20/78 | Kv1.1 K channel | pcDNA3.1 | HA sortase His | N100/13 | GST | pcDNA3.4 | HA sortase His |
| K28/38 | PSD-95 | pcDNA3.4 | HA sortase His | N103/31 | Aldh1L1 | pcDNA3.1 | sortase His |
| K28/42 | PSD-95  PSD-93 | pcDNA3.4 | HA sortase His | N103/31 | Aldh1L1 | pcDNA3.1 | HA sortase His |
| K28/43 | PSD-95 | pCDNA3.4 | HA His | N105/13 | Ankyrin-B | pcDNA3.4 | HA sortase His |
| K28/43 | PSD-95 | pcDNA3.1 | HA Flag sortase | N105/17 | Ankyrin-B | pcDNA3.4 | HA sortase His |
| K28/74 | PSD-95 | pcDNA3.4 | HA sortase His | N106/20 | Ankyrin-G | pcDNA3.4 | HA sortase His |
| K39/25 | Kv1.2 K channel (extracellular) | pCDNA3.4 | HA His | N114/10 | HCN4 | pcDNA3.1 | HA sortase His |
| K56A/50 | CASK | pcDNA3.4 | HA sortase His | N125/10 | Thorase/Atad1 | pcDNA3.4 | HA sortase His |
| K56A/57 | CASK | pcDNA3.4 | HA sortase His | N126B/31 | Neuregulin-CRD (Cysteine-rich domain, Type III) | pcDNA3.4 | HA sortase His |
| K60/73 | KChIP2b K channel | pcDNA3.4 | HA sortase His | N129/15 | Mad3 (human) | pcDNA3.4 | HA sortase His |
| K65/35 | CASPR/Neurexin IV | pcDNA3.1 | sortase His | N133/21 | RGS14 | pcDNA3.1 | sortase His |
| K66/27 | KChIP3 K channel | pcDNA3.4 | HA sortase His | N133/35 | RGS14 | pcDNA3.4 | HA sortase His |
| K66/38 | KChIP3 K channel | pcDNA3.4 | HA sortase His | N134/12 | Nav1.8 Na channel | pcDNA3.1 | sortase His |
| K66/59 | KChIP3 K channel | pcDNA3.4 | HA sortase His | N134/12 | Nav1.8 Na channel | pcDNA3.1 | HA sortase His |
| K89/34 | Kv2.1 K channel | pCDNA3.4 | HA His | N144/17 | Gamma-protocadherin-A3 | pcDNA3.4 | HA sortase His |

| | | | | | | | |
|---|---|---|---|---|---|---|---|
| K89/34 | Kv2.1 K channel | pcDNA3.1 | HA Flag sortase | N144/32 | Pan-Gamma-protocadherin-A | pcDNA3.4 | HA sortase His |
| K89/34 Flag | Kv2.1 K channel | pcDNA3.4 | HA His Flag | N149/25 | Olig1 | pcDNA3.4 | HA sortase His |
| K89/41 | Kv2.1 K channel | pcDNA3.4 | HA sortase His | N151/3 | GABA(A)R, Delta | pcDNA3.4 | HA sortase His |
| K96/7 | Pancortin | pcDNA3.4 | HA sortase His | N159/5 | Pan-Gamma-protocadherin-Constant | pcDNA3.4 | HA sortase His |
| L11A/41 | Pan-Neurofascin | pcDNA3.4 | HA sortase His | N160/21 | SynDIG3/Tmem91 | pcDNA3.4 | HA sortase His |
| L23/27 | Kv1.3 K channel | pcDNA3.1 | HA sortase His | N170A/1 | Neurexin-1-Beta | pcDNA3.1 | HA sortase His |
| L24/1 | IP3 receptor, type 1 | pcDNA3.4 | HA sortase His | N170A/26 | Neurexin-1-Beta | pcDNA3.4 | HA sortase His |
| L48A/9 | Cav1.3 Ca2 channel | pcDNA3.4 | HA sortase His | N183/15 | QKI-7 | pcDNA3.4 | HA sortase His |
| L61C/30 | Kv2.1 K channel | pcDNA3.4 | HA sortase His | N185/7 | NALCN | pcDNA3.4 | HA sortase His |
| L71/5 | Kv1.4 K channel (extracellular) | pcDNA3.4 | HA sortase His | N196/16 | PARIS/ZNF746 | pcDNA3.4 | HA sortase His |
| L76/36 | Kv1.2 K channel | pcDNA3.4 | HA sortase His | N201/35 | Iduna/RNF146 | pcDNA3.4 | HA sortase His |
| L83/11 | Kv2.1 K channel | pcDNA3.4 | HA sortase His | N206A/8 | GFAP | pcDNA3.1 | sortase His |
| L86/33 | AMIGO-1 | pcDNA3.4 | HA sortase His | N207/27 | LRP4 (extracellular) | pcDNA3.4 | HA sortase His |
| L107/39 | Neuroligin-2 | pcDNA3.1 | sortase His | N212/17 | TRIP8b (constant) | pcDNA3.4 | HA sortase His |
| L107/90 | Neuroligin-2 | pcDNA3.4 | HA sortase His | N221/17 | TRIP8b (constant) | pcDNA3.1 | sortase His |
| L107/95 | Neuroligin-2 | pcDNA3.4 | HA sortase His | N212A/34 | TRIP8b (exon 1b) | pcDNA3.4 | HA sortase His |
| L109/57 | Calbindin | pCDNA3.4 | HA His | N221/17 | TrpV1 | pcDNA3.4 | HA sortase His |
| L113/13 | Homer1L | pcDNA3.1 | sortase His | N225A/10 | FGF13/FHF2, B isoform | pcDNA3.4 | HA sortase His |
| L113/13 | Homer1L | pcDNA3.1 | HA sortase His | N241A/34 | LRRK2/Dardarin, C-terminus | pcDNA3.1 | sortase His |
| L113/130 | Homer1L/S | pcDNA3.1 | sortase His | N263/31 LC1 | Cav1.2 Ca2 channel | pCDNA3.4 | HA His |
| L113/130 | Homer1L/S | pcDNA3.1 | HA sortase His | N270/47 | Synaptotagmin-6 | pcDNA3.4 | HA sortase His |
| L113/27 | Homer1L | pcDNA3.1 | sortase His | N276A/15 | Synaptotagmin-9 | pcDNA3.4 | HA sortase His |
| L113/27 | Homer1L | pcDNA3.1 | HA sortase His | N289/16 | SUR1 | pcDNA3.4 | HA sortase His |

| | | | | | | | |
|---|---|---|---|---|---|---|---|
| L113/28 | Homer1L | pcDNA3.4 | HA sortase His | N295B/66 | Arl13b | pcDNA3.1 | HA His |
| L113/44 | Homer1L | pcDNA3.4 | HA sortase His | N355/1 | GluA1/GluR1 glutamate receptor | pcDNA3.1 | sortase His |
| L114/3 | Parvalbumin | pCDNA3.4 | HA His | N363/71 | Kir6.2 K channel | pcDNA3.4 | HA sortase His |
| L114/3 Flag | Parvalbumin | pcDNA3.4 | HA His Flag | N366/60 | Kir6.1 K channel | pcDNA3.4 | HA sortase His |
| L114/81 | Parvalbumin | pCDNA3.4 | HA His | N367/51 | Shank1 and Shank3 | pcDNA3.4 | HA sortase His |
| L115/13 | NPY/Neuropeptide Y | pcDNA3.1 | sortase His | N372B/1 | Kv2.2 K channel | pcDNA3.4 | HA sortase His |
| L115/13 | NPY/Neuropeptide Y | pcDNA3.1 | HA sortase His | N372B/60 | Kv2.2 K channel | pcDNA3.4 | HA sortase His |
| L117/1 | IRSp53/BAIAP2 | pcDNA3.1 | sortase His | N375/67 | Kv3.3 K channel | pcDNA3.4 | HA sortase His |
| L117/1 | IRSp53/BAIAP2 | pcDNA3.1 | HA sortase His | N393/2 | Beta4-spectrin | pcDNA3.4 | HA sortase His |
| L118/14 | VGAT | pcDNA3.4 | HA sortase His | N398A/34 | GABA(A)R, Alpha4 | pcDNA3.4 | HA sortase His |
| L120/12 | Collybistin | pcDNA3.1 | sortase His | N399/19 | GABA(A)R, Alpha2 | pcDNA3.4 | HA sortase His |
| L121/133 | nNOS/NOS1 | pcDNA3.4 | HA sortase His | N402/46 | Thyroid hormone receptor beta1 | pcDNA3.4 | HA sortase His |
| L121/136 | nNOS/NOS1 | pcDNA3.4 | HA sortase His | N403/63 | Pan-Thyroid hormone receptor | pcDNA3.4 | HA sortase His |
| L122/6 | Calretinin | pCDNA3.4 | HA His | N414/25 | NCKX4 | pcDNA3.4 | HA sortase His |
| L122/68 | Calretinin | pcDNA3.1 | sortase His | N422/18 | GluN2C/NR2C glutamate receptor | pcDNA3.4 | HA sortase His |
| L124/59 | Bassoon | pcDNA3.1 | sortase His | N432/21 | VSP | pcDNA3.4 | HA sortase His |
| L124/59 | Bassoon | pcDNA3.1 | HA sortase His | N432/63 | VSP | pcDNA3.4 | HA sortase His |
| L125/129 | Pan-Synapsin | pcDNA3.1 | sortase His | N448/88 | Kv8.2 K channel | pcDNA3.4 | HA sortase His |
| L127/8 | GAD67 | pcDNA3.1 | sortase His | N449/73 | VMAT2 | pcDNA3.4 | HA sortase His |
| L130/1 | Tiam1 | pcDNA3.4 | HA sortase His | N452/73 | GABA(A)R, Gamma2L/S | pcDNA3.1 | sortase His |
| L131/17 | SPHKAP | pcDNA3.4 | HA sortase His | N452/73 | GABA(A)R, Gamma2L/S | pcDNA3.1 | HA sortase His |
| L131/20 | SPHKAP | pcDNA3.4 | HA sortase His | N452/81 | GABA(A)R, Gamma2L/S | pcDNA3.1 | sortase His |
| L131/27 | SPHKAP | pcDNA3.4 | HA sortase His | N452/81 | GABA(A)R, Gamma2L/S | pcDNA3.1 | HA sortase His |

| | | | | | | | |
|---|---|---|---|---|---|---|---|
| N1/12 | KCC2 | pcDNA3.1 | HA sortase His | N471/27 | Kv1.7 K channel | pcDNA3.4 | HA sortase His |
| N16B/8 | Kv3.1b K channel | pcDNA3.1 | sortase His | N479/107 | VAPA/B | pcDNA3.4 | HA sortase His |
| N18/28 | PSD-93/Chapsyn-110 | pcDNA3.4 | HA sortase His | N479/12 | VAPA | pcDNA3.4 | HA sortase His |
| N22/21 | Shank1 | pcDNA3.4 | HA sortase His | N483/26 | Rufy3 | pcDNA3.4 | HA sortase His |
| N23B/6 | Shank2 | pcDNA3.4 | HA sortase His | N483/84 | Rufy3 | pcDNA3.4 | HA sortase His |
| N23B/49 | Pan-Shank | pcDNA3.1 | HA sortase His | N483/126 | Rufy3 | pcDNA3.1 | sortase His |
| N28/9 | VGlut1 | pcDNA3.1 | HA His | N483/126 | Rufy3 | pcDNA3.1 | HA sortase His |
| N28/9 Flag | VGlut1 | pcDNA3.4 | HA His Flag | N486/32 | c-Fos | pCDNA3.4 | HA His |
| N29/29 | VGlut2 | pcDNA3.1 | HA His | N486/76 | c-Fos | pcDNA3.4 | HA sortase His |
| N43/6 | Kv7.4/KCNQ4 K channel | pcDNA3.4 | HA sortase His | N112B/29 | Kir2.1 K+ channel | pcDNA3.4 | HA sortase His |
| N53/32 | BKbeta2 K channel | pcDNA3.4 | HA sortase His | N140A/12 | SALM5/LRFN5 | pcDNA3.4 | HA sortase His |
| N52A/42 | Mortalin/GRP75 | pcDNA3.1 | HA His | N155/9 | Uncx | pcDNA3.4 | HA sortase His |
| N52B/27 | SALM2/LRFN1 | pcDNA3.4 | HA sortase His | N294A/6 | Brevican | pcDNA3.4 | HA sortase His |
| N59/20 | GluN2B/NR2B glutamate receptor | pcDNA3.1 | sortase His | N312/10 | NSD1 | pcDNA3.4 | HA sortase His |
| N59/20 | GluN2B/NR2B glutamate receptor | pcDNA3.1 | HA sortase His | N411/51 | Arx | pcDNA3.4 | HA sortase His |
| N59/36 | GluN2B/NR2B glutamate receptor | pcDNA3.1 | HA sortase His | N416/57 | GluN3A/NR3A glutamate receptor | pcDNA3.4 | HA sortase His |
| N59/36 | GluN2B/NR2B glutamate receptor | pcDNA3.1 | HA His | N424/45 | Glycine receptor Alpha3 | pcDNA3.4 | HA sortase His |
| D4/154 | Kv2.1 K+ channel | pcDNA3.4 | HA sortase His | N428/12 | WRN | pcDNA3.4 | HA sortase His |
| K57/41 | Kv4.2 K+ channel (external) | pcDNA3.4 | HA sortase His | N452/30 | GABA-A-R-Gamma2L/S, GABRG2 | pcDNA3.4 | HA sortase His |
| L24/21 | IP3R | pcDNA3.4 | HA sortase His | N473/36.3 | Cavbeta3 Ca2+ channel | pcDNA3.4 | HA sortase His |
| L86/14 | AMIGO-1 | pcDNA3.4 | HA sortase His | N485/22 | Prrt2 | pcDNA3.4 | HA sortase His |
| L86/2 | AMIGO-1 | pcDNA3.4 | HA sortase His | **$V_L$-linker-$V_H$** | | | |
| L106/23 | Gephyrin | pcDNA3.4 | HA sortase His | L6/60 | Slo1/BKAlpha maxi-K channel | pcDNA3.4 | HA sortase His |

| | | | | | | | |
|---|---|---|---|---|---|---|---|
| L114/38 | Parvalbumin | pcDNA3.4 | HA sortase His | N86/8 | GFP | pcDNA3.4 | HA sortase His |
| L122/144 | Calretinin | pcDNA3.4 | HA sortase His | N95/35 | GABA(A)R, Alpha1 | pcDNA3.4 | HA sortase His |
| L123/46 | GABA(A)R, Theta | pcDNA3.4 | HA sortase His | N106/36 | Ankyrin-G | pcDNA3.4 | HA sortase His |
| N7/18 | Cavbeta1 Ca2+ channel | pcDNA3.4 | HA sortase His | N112B/14 | Kir2.1 K channel | pcDNA3.4 | HA sortase His |
| N56/21 | FGF14a | pcDNA3.4 | HA sortase His | N377/20 | MAP3K12 | pcDNA3.1 | HA sortase His |
| N112/16 | Kir2.1 K+ channel | pcDNA3.4 | HA sortase His | | | | |

**Supplementary Table 4.** List of scFvs and their targets that were successfully cloned into either pcDNA3.1 or pcDNA3.4 mammalian expression plasmids using the sequence-based Gibson Assembly cloning method. All are in the $V_H$-linker-$V_L$ format except as noted. Tags indicate the presence of HA or Flag epitope tags, a sortase labelling tag and/or the 6xHis purification tag at the C-terminus of the scFv.
